# Supplementary material for: Dog ecology and rabies knowledge, attitude and practice (KAP) in the Northern Communal Areas of Namibia
Source: PLoS Negl Trop Dis. 2024 Feb 5;18(2):e0011631. doi: 10.1371/journal.pntd.0011631 (PMC10881021; doi:10.1371/journal.pntd.0011631)
Supplement: S2 Table — (DOCX) [file pntd.0011631.s002.docx]

Supplementary table 2: Sociodemographic characteristics of the participants (n =3726)

| **Variable** | |  | **Frequency** | **Percentage** |
| --- | --- | --- | --- | --- |
| **Region** | Kavango East | | 455 | 12,21% |
|  | Kavango West | | 396 | 10,63% |
|  | Kunene | | 463 | 12,43% |
|  | Ohangwena | | 497 | 13,34% |
|  | Omusati | | 499 | 13,39% |
|  | Oshana | | 433 | 11,62% |
|  | Oshikoto | | 510 | 13,69% |
|  | Zambezi | | 473 | 12,69% |
| **Settlement** | Rural | | 2972 | 79,76% |
|  | Urban | | 754 | 20,24% |
| **Gender** | Female | | 1845 | 49,52% |
|  | Male | | 1881 | 50,48% |
| **Age** | 18-20 | | 138 | 3,70% |
|  | 21-24 | | 244 | 6,55% |
|  | 25-29 | | 372 | 9,98% |
|  | 30-34 | | 403 | 10,82% |
|  | 35-39 | | 447 | 12,00% |
|  | 40-44 | | 400 | 10,74% |
|  | 45-49 | | 372 | 9,98% |
|  | 50-54 | | 326 | 8,75% |
|  | 55-59 | | 290 | 7,78% |
|  | above 60 | | 734 | 19,70% |
| **Education** | Never attended school | | 809 | 21,71% |
|  | Primary level | | 974 | 26,14% |
|  | Secondary level | | 1568 | 42,08% |
|  | Graduate | | 375 | 10,06% |
| **Occupation** | Farmer | | 1568 | 42,08% |
|  | Unemployed | | 908 | 24,37% |
|  | Business | | 543 | 14,57% |
|  | Employee | | 473 | 12,69% |
|  | Student | | 234 | 6,28% |
| **Dog ownership** | no | | 1250 | 33,55% |
|  | yes | | 2476 | 66,45% |
| **Livestock ownership** | no | | 1286 | 34,51% |
|  | yes | | 2440 | 65,49% |
