# Supplementary material for: Dog ecology and rabies knowledge, attitude and practice (KAP) in the Northern Communal Areas of Namibia
Source: PLoS Negl Trop Dis. 2024 Feb 5;18(2):e0011631. doi: 10.1371/journal.pntd.0011631 (PMC10881021; doi:10.1371/journal.pntd.0011631)
Supplement: S3 Table — (DOCX) [file pntd.0011631.s003.docx]

Supplementary table 3: Number of dogs recorded during the survey and vaccinated against rabies in 2020 in different regions of the NCAs

| **region** |  | **DOHH** | | | |  | **number of dogs** | | | | | | | | | | |
| --- | --- | --- | --- | --- | --- | --- | --- | --- | --- | --- | --- | --- | --- | --- | --- | --- | --- |
|  |  |  | **vaccinated** | | |  | **Rural area** | | |  | **Urban area** | | |  | **Total** | | |
|  |  | **n** | **No** | **YES** | **%**  **vaccinated** |  | **n** | **vaccinated** | **%**  **vaccinated** |  | **n** | **vaccinated** | **%**  **vaccinated** |  | **n** | **vaccinated** | **%**  **vaccinated** |
| Kavango East |  | 294 | 188 | 106 | 36.1 |  | 505 | 123 | 24.4 |  | 233 | 110 | 47.2 |  | 738 | 233 | 31.6 |
| Kavango West |  | 263 | 152 | 111 | 42.2 |  | 573 | 167 | 29.1 |  | 94 | 46 | 48.9 |  | 667 | 213 | 31.9 |
| Kunene |  | 321 | 180 | 141 | 43.9 |  | 628 | 170 | 27.1 |  | 125 | 71 | 56.8 |  | 753 | 241 | 32.0 |
| Ohangwena |  | 329 | 136 | 193 | 58.7 |  | 602 | 276 | 45.8 |  | 67 | 34 | 50.7 |  | 669 | 310 | 46.3 |
| Omusati |  | 356 | 207 | 149 | 41.9 |  | 657 | 209 | 31.8 |  | 85 | 39 | 45.9 |  | 742 | 248 | 33.4 |
| Oshana |  | 308 | 96 | 212 | 68.8 |  | 550 | 302 | 54.9 |  | 95 | 66 | 69.5 |  | 645 | 368 | 57.1 |
| Oshikoto |  | 382 | 114 | 268 | 70.2 |  | 712 | 357 | 50.1 |  | 71 | 56 | 78.9 |  | 783 | 413 | 52.7 |
| Zambesi |  | 224 | 176 | 48 | 21.4 |  | 409 | 70 | 17.1 |  | 77 | 22 | 28.6 |  | 486 | 92 | 18.9 |
| total |  | 2477 | 1249 | 1228 | 49.6 |  | 4636 | 1674 | 36.1 |  | 847 | 444 | 52.4 |  | 5483 | 2118 | 38.6 |
