# Supplementary material for: Dog ecology and rabies knowledge, attitude and practice (KAP) in the Northern Communal Areas of Namibia
Source: PLoS Negl Trop Dis. 2024 Feb 5;18(2):e0011631. doi: 10.1371/journal.pntd.0011631 (PMC10881021; doi:10.1371/journal.pntd.0011631)
Supplement: S4 Table — (DOCX) [file pntd.0011631.s004.docx]

Supplementary Table 4: Univariable and final multivariable logistic regression model to determine the factors associated with dog vaccination among respondents of DOHHs (n=2476).

| **variables/categories** |  | **univariable model** | |  | **multivariable model** | |
| --- | --- | --- | --- | --- | --- | --- |
|  |  | **OR (95% CI)** | **P-value** |  | **Adj.OR (95% CI)** | **P-value** |
| **respondent residence** | | | | | | |
| rural |  | reference |  |  | reference |  |
| urban |  | 1.7 (1.4-2.1) | <0.001 |  | 2.7 (2.1-3.6) | <0.001 |
| **Respondent region** |  |  |  |  |  |  |
| Kavango East |  | reference |  |  | reference |  |
| Kavango West |  | 1.2 (0.9-1.8) | 0.137 |  | 1.2 (0.8-1.7) | 0.377 |
| Kunene region |  | 1.4 (1.0-1.9) | 0.046 |  | 1.3 (0.9-1.8) | 0.131 |
| Ohangwena |  | 2.5 (1.8-3.5) | <0.001 |  | 2.8 (1.9-3.4) | <0.001 |
| Omusati |  | 1.3 (0.9-1.7) | 0.132 |  | 1.4 (1.1-2.0) | 0.032 |
| Oshana |  | 3.9 (2.8-5.5) | <0.001 |  | 3.4 (2.7-5.5) | <0.001 |
| Oshikoto |  | 4.1 (3.0-5.7) | <0.001 |  | 4.9 (3.4-6.9) | <0.001 |
| Zambezi |  | 0.5 (0.3-0.7) | 0.0002 |  | 0.5 (0.3-0.7) | 0.0008 |
| **livestock ownership status** | | | | | | |
| no |  | reference |  |  | reference |  |
| yes |  | 1.4 (1.2-1.7) | 0.0002 |  | 2.1 (1.7-2.7) | <0.001 |
| **respondent gender** | | | | | | |
| female |  | reference |  |  | reference |  |
| male |  | 1.4 (1.2-1.6) | <0.001 |  | 1.6 (1.2-1.8) | <0.001 |
| **having heard of rabies** | | | | | | |
| no |  | reference |  |  | reference |  |
| yes |  | 2.5 (1.8-3.4) | <0.001 |  | 2.1 (1.7-2.7) | 0.017 |
