# Supplementary material for: Dog ecology and rabies knowledge, attitude and practice (KAP) in the Northern Communal Areas of Namibia
Source: PLoS Negl Trop Dis. 2024 Feb 5;18(2):e0011631. doi: 10.1371/journal.pntd.0011631 (PMC10881021; doi:10.1371/journal.pntd.0011631)
Supplement: S5 Table — (DOCX) [file pntd.0011631.s005.docx]

Supplementary Table 5: Dog bite reporting and resulting dog bite incidences for the regions of the NCAs.

|  | **respondents declared** | |  |  |
| --- | --- | --- | --- | --- |
| **region** | **dog bite** | **No dog bite** | **surveyed population** | **dog bite incidence/ 100,000 (95%CI)*** |
|  |  |  |  |  |
| **Kavango East** | 97 | 358 | 4,006 | 1,210 (950-1,500) |
| **Kavango West** | 35 | 361 | 3,434 | 510 (367-707) |
| **Kunene** | 24 | 439 | 4,577 | 262 (176-390) |
| **Ohangwena** | 66 | 431 | 4,658 | 708 (558-899) |
| **Omusati** | 23 | 476 | 3,369 | 341 (228-511) |
| **Oshikoto** | 28 | 482 | 3,508 | 399 (276-576) |
| **Zambezi** | 76 | 397 | 2,775 | 1,369 (1,097-1,707) |
| **Total NCA** | 403 | 3,323 | 29,892 | 674 (612-743) |

* based on 2019 census data
