# Supplementary material for: Dog ecology and rabies knowledge, attitude and practice (KAP) in the Northern Communal Areas of Namibia
Source: PLoS Negl Trop Dis. 2024 Feb 5;18(2):e0011631. doi: 10.1371/journal.pntd.0011631 (PMC10881021; doi:10.1371/journal.pntd.0011631)
Supplement: S6 Table — (DOCX) [file pntd.0011631.s006.docx]

Supplementary table 6. Univariable and final multivariable logistic regression model to determine the factors associated with the respondent knowledge of rabies (good vs fair to poor) in NCA, Namibia (2021).

| **variables/categories** |  | **univariable model** | |  | **multivariable model** | |
| --- | --- | --- | --- | --- | --- | --- |
|  |  | **OR (95% CI)** | **P-value** |  | **Adj.OR (95% CI)** | **P-value** |
| **gender** |  |  |  |  |  |  |
| female |  | 1 |  |  | 1 |  |
| male |  | 1.4 (1.2 - 1.6) | <0.001 |  | 1.5 (1.3 - 1.7) | <0.001 |
| **respondent education level** | | | | | | |
| attended school |  | 1 |  |  | 1 |  |
| never attended school |  | 0.7 (0.6 - 0.8) | 0.0007 |  | 0.7 (0.6 - 0.9) | 0.0007 |
| **respondent residence** |  |  |  |  |  |  |
| rural |  | 1 |  |  | 1 |  |
| urban |  | 1.6 (1.4 - 2.0) | <0.001 |  | 1.7 (1.4 - 2.0) | <0.001 |
| **dog ownership status** |  |  |  |  |  |  |
| no |  | 1 |  |  | 1 |  |
| yes |  | 1.2 (1.0 - 1.3) | 0.047 |  | 1.2 (1.1 - 1.4) | 0.016 |
