# Supplementary material for: Dog ecology and rabies knowledge, attitude and practice (KAP) in the Northern Communal Areas of Namibia
Source: PLoS Negl Trop Dis. 2024 Feb 5;18(2):e0011631. doi: 10.1371/journal.pntd.0011631 (PMC10881021; doi:10.1371/journal.pntd.0011631)
Supplement: S7 Table — (DOCX) [file pntd.0011631.s007.docx]

Supplementary table 7. Final multivariable logistic regression model to determine the factors associated with the respondent attitude towards rabies (favourable vs unfavourable attitude) in NCA, Namibia (n=3252)

| **variables/categories** |  | **univariable model** | |  | **multivariable model** | |
| --- | --- | --- | --- | --- | --- | --- |
|  |  | **OR (95% CI)** | **P-value** |  | **Adj.OR (95% CI)** | **P-value** |
| **respondent education level** |  |  |  |  |  |  |
| attended school |  | reference |  |  | reference |  |
| never attended school |  | 0.6 (0.5-0.7) | <0.001 |  | 1.4 (1.8-2.3) | <0.001 |
| **respondent occupation** |  |  |  |  |  |  |
| business |  | reference |  |  | reference |  |
| employee (govt/corporate) |  | 1.2 (0.9-1.5) | 0.235 |  |  |  |
| farmer |  | 0.6 (0.5-0.8) | <0.001 |  |  |  |
| student |  | 1.7 (1.2-2.3) | 0.002 |  |  |  |
| unemployed |  | 0.5 (0.4-0.6) | <0.001 |  |  |  |
| **respondent residence** |  |  |  |  |  |  |
| rural |  | reference |  |  | reference |  |
| urban |  | 2.1 (1.8 - 2.5) | <0.001 |  | 1.8 (1.5 - 2.2) | <0.001 |
| **dog ownership status** |  |  |  |  |  |  |
| no |  | reference |  |  | reference |  |
| yes |  | 0.9 (0.8 - 1.0) | 0.057 |  |  |  |
| **rabies knowledge score** |  |  |  |  |  |  |
| below mean score (12.6) |  | reference |  |  | reference |  |
| above mean score (12.6) |  | 2.7 (2.3 - 3.0) | <0.001 |  | 2.5 (2.2 - 2.9) | <0.001 |
