# Supplementary material for: Dog ecology and rabies knowledge, attitude and practice (KAP) in the Northern Communal Areas of Namibia
Source: PLoS Negl Trop Dis. 2024 Feb 5;18(2):e0011631. doi: 10.1371/journal.pntd.0011631 (PMC10881021; doi:10.1371/journal.pntd.0011631)
Supplement: S8 Table — (DOCX) [file pntd.0011631.s008.docx]

Supplementary table 8: Respondents' statements regarding traditional treatment of dog bites and treatment of dogs bitten by other dogs (by region) in NCAs (n=3726)

| **Traditional treatment** | **Kavango East** | **Kavango West** | **Kunene** | **Ohangwena** | **Omusati** | **Oshana** | **Oshikoto** | **Zambezi** | **Total** | **Percent** |
| --- | --- | --- | --- | --- | --- | --- | --- | --- | --- | --- |
| No | 318 | 321 | 289 | 255 | 285 | 277 | 328 | 307 | 2380 | 63.9 |
| Unknown | 110 | 63 | 158 | 177 | 172 | 104 | 143 | 121 | 1048 | 28.1 |
| Yes | 27 | 12 | 16 | 65 | 42 | 52 | 39 | 45 | 298 | 8 |
