# Supplementary figures and images for: Dog ecology and rabies knowledge, attitude and practice (KAP) in the Northern Communal Areas of Namibia
Source: PLoS Negl Trop Dis. 2024 Feb 5;18(2):e0011631. doi: 10.1371/journal.pntd.0011631 (PMC10881021; doi:10.1371/journal.pntd.0011631)

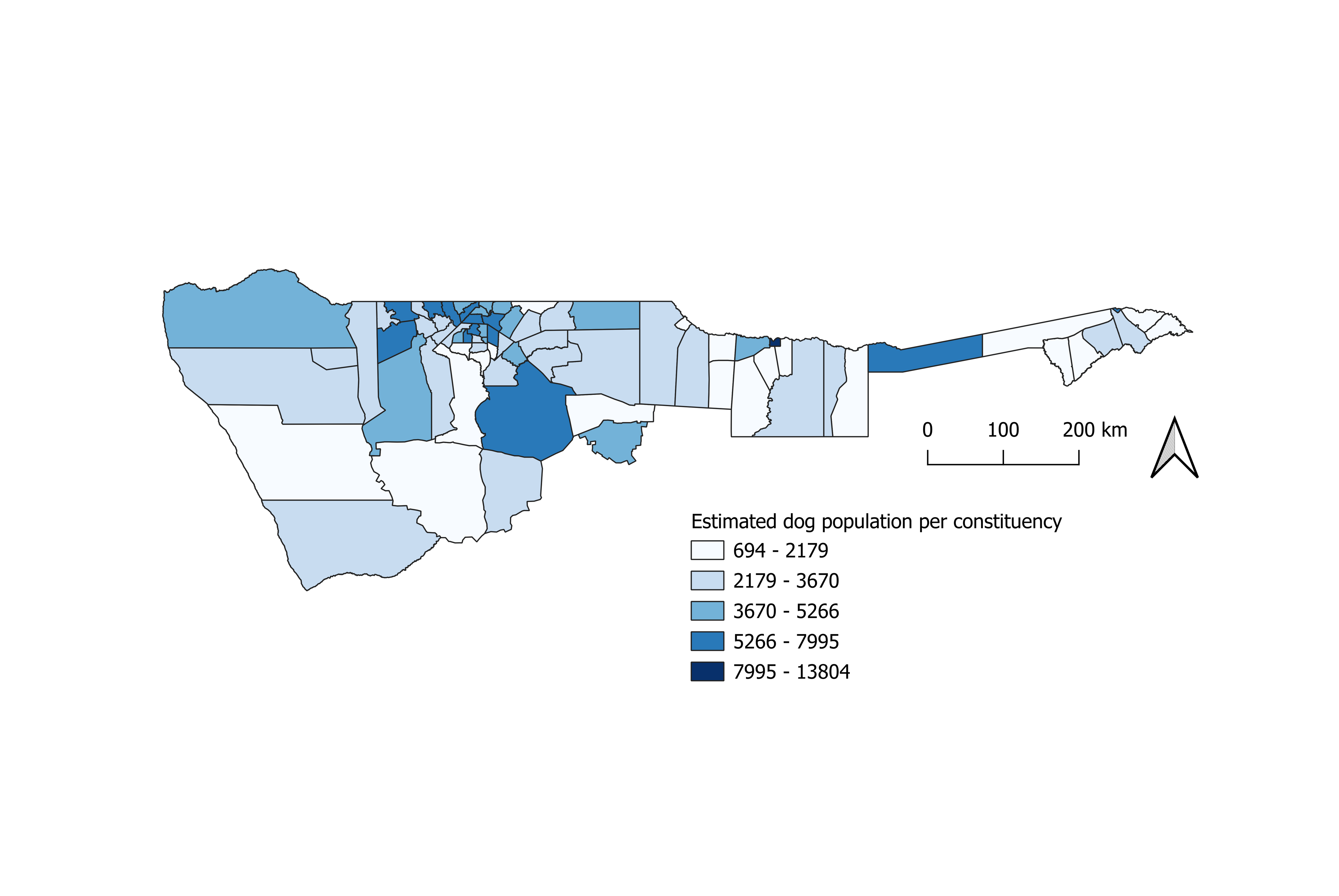

Supplement: S1 Fig — Map content was produced with Esri ArcGIS software using study data and data provided by GADM available online: https://gadm.org/download_country.html. (TIF) [file pntd.0011631.s009.tif]

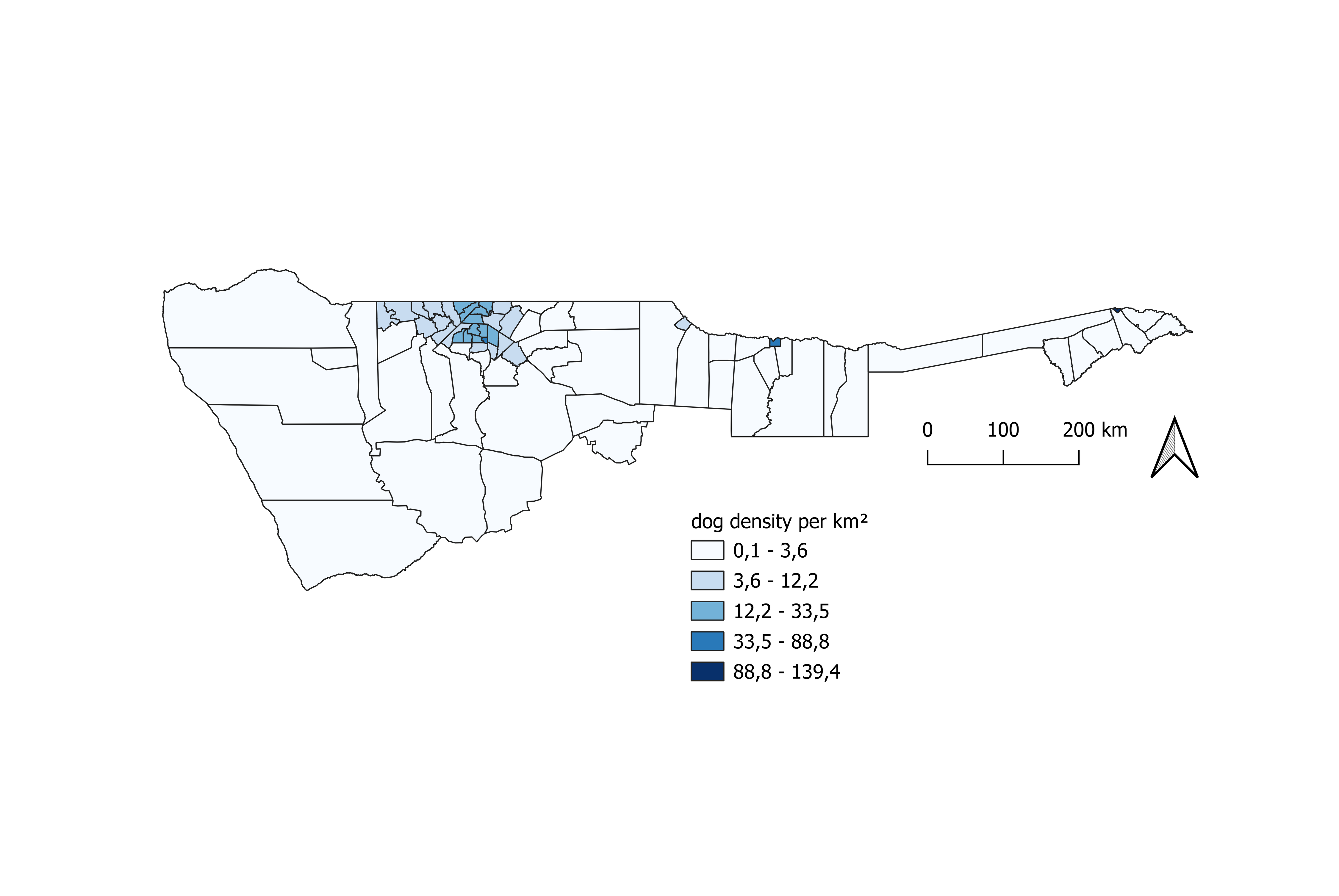

Supplement: S2 Fig — Map content was produced with Esri ArcGIS software using study data and data provided by GADM available online: https://gadm.org/download_country.html. (TIF) [file pntd.0011631.s010.tif]
