# Supplementary material for: Dog ecology and rabies knowledge, attitude and practice (KAP) in the Northern Communal Areas of Namibia
Source: PLoS Negl Trop Dis. 2024 Feb 5;18(2):e0011631. doi: 10.1371/journal.pntd.0011631 (PMC10881021; doi:10.1371/journal.pntd.0011631)
Supplement: Attachment — Submitted filename: Point-by-point response.docx [file pntd.0011631.s011.docx]

**Point-by-point response**

The authors thank the three reviewers for their constructive comments and suggestions that helped to improve this manuscript.

**Methods**

Reviewer #1: The methods are clearly described with sufficient detail to be replicated by others. They provide the required information to design similar studies and to explain the approach that the authors used to obtain the results.

Reviewer #2: The study objectives are clearly articulated, and the rationale well explained. Clear that this KAP survey will fill a gap in knowledge in this area and it is a well utilised and accepted survey tool in this context. Is there any evidence that KAP surveys elsewhere have translated into better rabies control programs?

Response:

This is a legitimate question for which we have no definite answer. One assumes that those KAP studies are sort of eye openers for responsible authorities, but after re-reviewing the literature, unfortunately, there does not appear to be any work claiming that KAP surveys have really led to better rabies control programs elsewhere. At least where improvements are described, no reference is made to previous KAP studies.

Population characteristics well described.

Methods: If the crush pens are used for dog vaccination program, there seems potential for bias here in that houses in close proximity may have different KAP to those far away.

Response:

Chrush pens are located differently within communities, and given the numbers of crush pen chosen we believe that any potential bias would be negligible.

The survey data collection and analysis methods seems sound. No ethical concerns.

Reviewer #3: The methods are clearly articulated and appropriate for this study. However I could not find the questionnaire which is said to be available in the supplementary materials.

Response:

The questionnaire was again uploaded as supplementary material.

**Results**

Reviewer #1: The results were clearly presented in a logical manner that was easy to follow. The tables, figures and maps were well presented and clear.

Reviewer #2: Demographics and geographical area clearly presented. (No units for age in Fig 2).

Response:

Age units were included in the revised version.

The urban vs rural results are interesting. Some of the graphs are hard to read because of the small text and questions being described in titles, but key results are pulled out in text.

Response:

All figures were provided as high-resolution images as requested as per authors instructions. So, when included during the production process the readability should be much better.

Fig 7- are Kavango east and Zambezi different in any way to other regions? urban/rural?

Response:

Neither in our data nor in the latest data from the Namibian Statistical Agency are there any significant differences between Zambezi and Kavango East that would explain the high dog bite incidences. This was added to the discussion.

Reviewer #3:

**Conclusions**

Reviewer #1: The conclusions adequately addressed the results, while also considering any potential shortcomings related to the study design and methodology. Discrepancies and results of particular interest were highlighted and explained further with additional considerations, making the conclusions clear and well-articulated.

Reviewer #2: There is a strong argument for understanding the dog demographics leading to a better planned dog vaccination program. However, I don't think the detail of how to get the vaccine coverage rate higher has really been addressed- for those that didn't vaccinate their dogs, does the survey give any insights into why? Is this access or attitudes mainly? This is touched on in lines 505/506 but I think needs to be expanded.

Response:

We thank the reviewer for bringing this to our attention. The question of increasing vaccination coverage in dogs has already been partially answered in the paragraph below. However, we have expanded on the question and changed the text in the revised version by including new references as follows: " Therefore, to increase vaccination coverage in dogs future MDV campaigns in NCAs will need to adjust the number of vaccine doses to match the dog population per constituency identified in this study, ideally with an upward safety margin, while reconsidering the number and strategic selection of locations for vaccination sites considering landscape and topography [71]. Monitoring MDV campaigns using specially designed vaccination devices for data collection and subsequent GIS analysis using gridded population data can help to better assess and, if necessary, improve vaccination coverage at the local level [9]. In view of the high proportion of hard-to-reach dogs and Namibia's extremely positive experience with the efficiency of oral vaccination of dogs from field trials [43,72], the long-term strategic integration of this vaccination variant into the national dog rabies control program should be seriously considered. Also, the fact that nearly…”

The bite behavior is interesting and shows a high take up of PEP, which is encouraging. The conclusion draws together some suggestions how the authors think these results can specifically be used to improve the vaccination program and increase coverage rates, to help eliminate rabies.

Reviewer #3: The conclusions and limitations are completely described. The authors make sufficient mention of the wider application of the findings.

**Editorial and Data Presentation Modifications**

Reviewer #1: Please note that the questionnaire was not available in the supplementary materials (at least to the reviewer). Please be sure to include this in the final manuscript as this would be something of importance to others looking to replicate the study elsewhere. Throughout the manuscript the authors switch terminology between “dog” and “canine”. Please standardize for clarity.

Response:

We have consistently used one term as requested.

Please check the punctuation after abbreviations throughout the manuscript, for example when using “e.g.” please include a comma after the abbreviation. “e.g.,” (line 137 and elsewhere).

Response:

Commata were included after abbreviations as requested.

Additional minor comments below should be considered for revision.

Line 61: replace “on” with “in”

Line 71: “need” should be “needs”

Line 89: Please either change the “but” to "and" or say "vast majority is free-roaming, not only supporting disease transmission but further complicating control efforts."

Line 104: delete “organization” – it should be referred as the tripartite only.

Line 151: spelling: integrated.

Line 164: double space between last word and reference.

Line 199: Spelling: Respondent

Line 231: “according to…” – the word “to” is missing.

Line 231: Spelling: Agency

Line 276: Figure 1 caption “… June 2021 is shown” – the word “is” is missing.

Line 284: The range of people living in a household was 1 – 70 people. Is this correct? Was one household found to have 70 people living there?

Yes, this was recorded during the study.

Line 284: Please define the age of a “child” – is this anyone below 18 years of age?

Line 294: Spelling: DOHHs

Line 301: HDR mentioned is 1:5.45 – please remove the “1:” as this implies one person for every 5.45 dogs.

Line 324: “Oshikato are supposed to have dog densities higher than 30 dogs/km²” – this statement is unclear. Why are they “supposed” to have those densities? Why are the areas with far higher densities not mentioned?

Line 380: Figure 6 caption: “Answers of respondents when ask what would they do if they” – consider revising to "when asked what they would do…"

Line 380: Figure 6 caption: “(A) would encounter” - consider changing to “encountered”

Line 410: “behavior in case of bite exposure” – consider revising to “behavior in the case of bite exposure” (include the word “the”).

Line 450 – 451: “despite a national rabies control strategy in place” – a verb is missing. Consider revising to “despite a national rabies control strategy already being in place…”

Line 518: Change “rabies” to “rabid”

Supporting information, Table S2 description: “the” is repeated accidentally.

S1 Figure description: “constituency wise” should change to “constituency wide” Alternatively, a preferred modification would be "showing the estimated dog population per constituency based…"

S2 Figure description: “constituency wise” should change to “constituency wide” Alternatively, a preferred modification would be "showing the estimated dog density per constituency."

Line 670 (references): Double punctuation after “2015”.

Line 854: The first author name is represented incorrectly. Change to “De Balogh KK,”

Response:

Thank you for the detailed review; all proposals above were accepted in the revised version

Reviewer #2: The author summary reads much like a technical summary rather than a non-technical summary and seems too repetitive of content within the abstract. I would suggest rewriting this with the 'non-technical' brief in mind and using plainer language. Eg omit One Health, "Our results show that there was 1 dog for about every 5 humans". etcLine 151- typo "intergated" should be integrated. Inconsistent decimal points for proportions have been presented, sometimes it is 1 and sometimes 2.

Response:

The reviewer is right. We have rewritten the authors summary accordingly.

“As a neglected disease, rabies remains a major problem in Africa and Asia. Here we report the results of an extensive community survey on dog ownership and knowledge, attitudes and practices (KAP) related to rabies control and prevention, covering the Northern Communal Areas (NCAs) of Namibia. The survey conducted in 2021 included more than 3,700 households and provided useful insights that will inform interventions in order to improve human rabies prevention and Namibia’s dog rabies control strategy. Our results show that there was 1 dog for about every 5 humans, of which the vast majority is free-roaming. This surprisingly high dog population is not only supporting disease transmission but further complicating control efforts. Most people were aware that dog-mediated rabies is present in this part of Namibia and had an acceptable attitude and behavior towards it. Depending on the region, between 262 and 1,369 people per 100,000 inhabitants were bitten by dogs during the survey period. Although the number of dog bites is relatively high, more than 90% of victims stated that they had sought hospital treatment after being bitten. However, there are still gaps in laboratory-based surveillance of dog-mediated rabies, and incomplete or lack of rabies prophylaxis after dog bite injuries have led to unreported human deaths. Therefore, improved vaccination measures for dogs, consistent rabies prophylaxis after dog bite injuries as well as awareness-raising measures to increase people's knowledge and awareness are necessary if human deaths caused by rabies are to be permanently prevented”

Reviewer #3: Abstract typos/minor modifications:

"will inform interventions on..." - should read will inform interventions in..."

"revealed deficiencies in some of the population." - should be reworded for clarify, perhaps "revealed deficiencies in knowledge, in some of the population."

Main text typos/modifications:

Line 104: (WHO, WOAH, and FAO) need to be defined

Line 230: "calculated projected" are both of these words necessary?

Line 439-440: "significant time efforts" - needs to be reworded for clarity , suggest "significant time and effort" ??

Line 440: (n=18) Is this the number of teams, individuals in teams or days working?

Response:

Thank you for the detailed review; all proposals above were accepted in the revised version

Summary and General Comments

Reviewer #1: The study provided a detailed description and the results from a combined KAP survey and dog population estimate study, reaching an impressively large number of households in the at-risk areas for rabies in Namibia. Results indicated an inferior level of knowledge and attitudes towards rabies, with a need to also improve practices further. The dog population was estimated using the HDR with a value of 5.45 resulting in a total estimated population of approximately ~240 000 - 270 000 dogs in the NCAs. The study was well conceived and well-presented and provides important information to the elimination efforts in Namibia. It also provides good insights and information for others to replicate the study in other rabies-endemic regions around the world.

Reviewer #2: This paper is well written and gives a comprehensive account of this important study in Namibia. The insights gained will help improve prevention measures if implemented. This clearly has high significance for Namibia, but I am less clear on the originality of the paper and the usefulness of the findings globally. However, other communities can learn from these findings and some of the themes and suggestions for program improvements are clearly transferable.

Response:

The reviewer is right. We have modified the Conclusion section as follows: “…if substantial progress is to be made in the control of dog-mediated rabies in the near future. The results are obviously of great importance to Namibia, however, other communities in Africa can learn from these findings and some of the themes and suggestions for rabies control program improvements are certainly clearly transferable.”

Reviewer #3: Overall, this is a well written manuscript with important results applicable for rabies control.
